# Characterisation of soils under long-term crop cultivation without fertilisers: a case study in Japan

**DOI:** 10.1186/s40064-016-1917-y

**Published:** 2016-03-05

**Authors:** Hiroko Nakatsuka, Kenji Tamura

**Affiliations:** Graduate School of Life and Environmental Sciences, University of Tsukuba, Tsukuba, Japan; Faculty of Life and Environmental Sciences, University of Tsukuba, Tsukuba, Japan

**Keywords:** Soil, Micromorphology, Fractal dimension, Physicochemical properties, Unfertilised farming system, Organic farming, Andosols

## Abstract

Certain farms in Japan, namely unfertilised farms (UFs), have been able to maintain high productivity for over 40 years without applying fertilisers or composts. This study aimed to characterise the physicochemical, biological and micromorphological properties of soil in UFs compared with control farms in Eniwa and Nariita and to identify characteristics that are associated with crop productivity. In UFs, no plough pan was observed. The thickness of the effective soil depth (ESD) of UFs was greater than that of CFs. The concentrations of soil organic carbon, total nitrogen and nitrate-nitrogen in ESD of UFs were higher than those in ESD of CFs. Soil microstructure observations indicated the strong development of a granular microstructure with large amounts of void space and a high fractal dimension in both surface and subsoil horizons of UFs. Dry yield had a strong correlation with ESD thickness and fractal dimension of voids. Thus, the management of unfertilised cultivation promoted the development of soil aggregation in both A and B horizons. The increase in ESD, soil pore spaces and complexity with the development of subsoil structure improved the productivity of unfertilised cultivation.

## Background

Problems such as groundwater and river nitrogen pollution have occurred in Japan (Hojito et al. 2003; Kumazawa 1999; Yamaki et al. 2013), and sustainable low-intensity agriculture is attracting increasing attention (Mander 1999). To address this issue, unfertilised cultivation, an organic farming systems featuring cultivation without the use of fertilisers, has become popular in Japan (Oda and Hosen 2011; Yoshima 2005). This type of cultivation aims to produce crops without using any fertiliser or compost so as to exploit fully a soil’s capacity for crop production (Okada 1953). The specific method is as follows: (1) no agricultural chemicals, fertilisers or manures (nitrogen, phosphorus or potassium fertilisers, composts, animal manure, minerals, etc.) are applied; (2) in-house seeds are used; (3) continuous cropping of the same crop is maintained; (4) the soil surface is covered to a depth of approximately 1 cm with mulch, which incorporated to a depth of approximately 10 cm using a rotary tiller after harvesting and (5) weeding is done two or three times per year (Ishii 2010; Oda and Hosen 2011). This cultivation system leads to lower environmental impacts and reduced input costs (Ishii 2010; Oda and Hosen 2011). Several small-scale trials of this method have been attempted. However, plants grown without fertilisers generally have 20–30 % lower yields than fertilised conventional farming systems (Yoshida 1987). In special cases, high-yield farms have been cultivated without fertilisers (unfertilised farms; UFs) and with continuous cropping for over 40 years in Eniwa (Hokkaido) and Narita (Chiba prefecture), Japan. These UFs produce normal amounts of crops for marketing without application of fertilisers or composts, applying only organic materials as mulch (sources can include rice or wheat straw, weeds, cornstalks etc.). Mulch is obtained from outside the farm; it is not composted or supplemented with minerals or nutrients. A study of the effect of soil characteristics on productivity in those farms may give pointers for increasing crop productivity in low-input agriculture.

Under unfertilised cultivation, total soil carbon and nitrogen contents of soil reached equilibrium after 19 years (Kuwada et al. 2006). Oda and Hosen (2011) determined the source of nitrogen in tomato leaves that were cultivated on an UF using the δ^15^N method, which suggested that a substantial amount of N with relatively low δ^15^N values (such as atmospheric N) was received from outside the field–plant system, and this was believed to improve crop production over that observed on an UF. Thus, the productivity of UFs is affected by some biological activities such as nitrogen fixation.

In general, soil biological activities are associated with soil structure (Rillig and Mummey 2006), which in turn affects root penetration and the movement of water and gases. Yoshino (1993) reported that solid ratio and aggregate stability increased in the soil surface on UFs, suggesting that the physical properties of soil are improved by unfertilised farming systems. We hypothesized that improving the physical and biological properties of soil would increase crop yield in high-yield UFs. To test this hypothesis, we studied the physicochemical, biological and morphological properties of soils in long-term UFs in Japan. In general, biological activities are influenced by chemical fertilisers and organic manures (Marschner et al. 2003). Moreover, soil structure, particularly soil aggregates, is influenced by the tillage method (Pagliai et al. 2004; Plante and McGill 2002; Six et al. 1999). We investigated the effects of shallow tillage and unfertilised management of unfertilised farming systems and compared them with the effects of deep tillage of farm with no chemical fertiliser (Eniwa, Hokkaido) and those of a shallow-tillage farm with chemical fertilisers (Narita, Chiba). The objectives of the present study were to determine the soil characteristics of UFs and to identify characteristics that are associated with crop productivity.

## Methods

### Site descriptions and farm management

This study investigated UFs and control farms (CFs) in Eniwa, Hokkaido (E-) and Narita, Chiba (N-), belonging to local farmers. The climate is classified as humid continental at Eniwa and humid subtropical at Narita according to the Köppen–Geiger climate classification (Climate-Data.Org 2015a, b). The average annual rainfall is 1044 mm at Eniwa and 1546 mm at Narita and the annual total snow depth is 5760 mm in Eniwa, with snow occurring mainly from November to March (Japan Meteorological Agency 2015a, b). The study farms are flat and the soils are formed of volcanic ash. According to the World Reference Base for Soil Resources (WRB; IUSS Working Group 2014), E-UF and E-CF are classified as vitric andosols and N-UF and N-CF are classified as silandic andosols (Table 1).

**Table 1:** The cropping and management history of the Eniwa and Narita farms

	Eniwa		Narita	
	Unfertilised farm	Control farm	Unfertilised farm	Control farm
Soil classification (WRB)	Vitric andosol	Vitric andosol	Silandic andosol	Silandic andosol
Unfertilised cultivation history (year)	56	–	41	–
Farm area (ha)	0.18	0.10	0.25	0.20
Tillage depth (cm)	10	40	10	20
Implements used	Rotary tiller	Mouldboard plough and rotary tiller	Rotary tiller	Rotary tiller
Mulch type	Weed and plant residue	Plant residue	Cornstalk	Composted fallen leaves
Mulch rates (dry) (kg ha^−1)^	0.4	0.3	0.4	1.0
Nutrient inputs in mulch	TC: 35.0 %, TN: 2.4 %, C/N: 15, moisture: 41.6 %	TC: 35.3 %, TN: 1.9 %, C/N: 18, moisture: 80.3 %	TC: 38.9 %, TN: 1.7 %, C/N: 23, moisture: 59.6 %	TC: 35.8 %, TN: 2.0 %, C/N: 18, moisture: 27.2 %
Chemical fertilisers	Not use	Not use	Not use	N,P,K
Input of chemical fertilisers (t ha^−1^)	0	0	0	0.5
Seed type	House seeds	Commercial seedling	House seeds	Commercial seeds

The farm areas were approximately 0.10–0.25 ha in four farms (Table 1). In total, approximately 115 vegetable crops were cultivated on E-UF and 60 on N-UF. The specific method of UFs had been applied for 56 years in Eniwa and for 41 years in Narita (Table 1). CFs was adjacent to UFs. E-CF used organic manure but did not use pesticides, weed killers or chemical fertilisers. E-CF was tilled to a depth of approximately 40 cm by a mouldboard plough and rotary tiller (Table 1). N-CF used chemical and organic manures, but not pesticides or weed killers, and the tillage depth was 20 cm (using only a rotary tiller) (Table 1). Commercially available seedlings and seeds were used on CFs.

### Crop yield

Mean dry yield was determined in the growing season (October 2012, tomato; May 2012, green onion) by quantifying the peak dry mass of plants per unit area at each farm. Multiple stations for each treatment were sampled to determine plant biomass by clipping plants within areas of 60 × 80 cm^2^ on Eniwa farms and 30 × 30 cm^2^ on Narita farms. After cutting, all of the plants produced on the farms were separated and dried at 70 °C for 3 days. The yield is reported on a dry weight basis.

### Observation of soil profile morphology and sample collection

Soil surveys were conducted in farms cultivated with tomatoes (*Solanum lycopersicum*) on E-UF (E 141°35′35.44″, N 42°52′17.85″) and courgettes (*Cucurbita pepo*) on E-CF (E 141°35′35.62″, N 42°52′17.76″) in October 2011, with green onion (*Allium fistulosum*) on N-UF (E 140°20′35.10″, N 35°46′34.80″) in June 2010 and after sweet potato (*Ipomoea batatas*) cultivation on N-CF (E 140°20′33.00″, N 35°46′33.86″) in April 2011. The distance in a straight line between the soil profiles of E-UF and E-CF is approximately 5 m and that of N-UF and N-CF is approximately 50 m. The soil profiles were assigned according to the United Soil Classification System of Japan (The Fourth Committee for Soil Classification and Nomenclature in the Japanese Society of Pedology 2010). Soil samples were collected from each horizon, and air-dried soils passed through 2 or 0.5 mm sieves used for chemical analysis. Soil core samples were collected from the four farms using a 100 mL steel corer (5 cm in diameter) at depths of 0–5, 10–15, 30–35, 50–55 and 70–75 cm from E-UF and N-UF; 0–5, 12–17, 30–35, 50–55 and 70–75 cm from E-CF and 0–5; 20–25, 35–40, 50–55 and 70–75 cm from N-CF to determine the physical properties (taking three core samples from each horizon) and to prepare thin sections (taking one core sample for each horizon) of the soil. For biological analysis, fresh soil was sampled from the surface (0–5 cm) and subsurface (15–20 cm) in farms cultivated with tomatoes (*S. lycopersicum*) in E-UF and E-CF in October 2011 and with green onions (*A. fistulosum*) in N-UF and N-CF in October 2011. Soil samples were sieved to 2 mm in a field-moist condition and stored at 4 °C before biological analysis.

### Analytical methods for determining physicochemical properties

Soil physical properties were analysed using three soil core samples. Bulk density (Blake and Hartge 1986) and saturated hydraulic conductivity (SHC) were measured by the SC method (Lee et al. 1985). The three-phase ratio and porosity were measured according to Committee of Soil Environmental Analysis (1970) and Hillel (1998). First, we estimated the effective volumetric capacity using an effective volumetric capacity analyser (DIK-1150, Daiki). Next, the samples were saturated with degassed water for 48 h and weighed (*W*_1_). SHC was then determined according to the falling head test method. Finally, the samples were dried at 105 °C for 24 h and weighed (*W*_2_). We calculated the bulk density, three-phase ratio (solid, liquid and gaseous ratios) and porosity using the results obtained. Porosity distinguishes between micro pores (P_mi_) and macro pores (P_ma_). P_mi_ was calculated by Eq. (1) and P_ma_ was calculated by Eq. (2).

1 $${\text{P}}_{\text{mi}} = W_{1} - W_{2}$$

2 $${\text{P}}_{\text{ma}} = ({\text{liquid}}\,{\text{and}}\,{\text{gaseous}}\,{\text{ratios}}) - {\text{P}}_{\text{mi}}$$

Soil chemical properties were determined by standard methods. Each soil chemical analysis was replicated two times. Soil pH(H_2_O) and pH(KCl) (1:2.5 soil–water suspensions) were measured according to Thomas (1996) and pH(NaF) were determined according to Van Reeuwijk (2002). Organic carbon content (OC) and total nitrogen content (TN) were measured using an NC analyser (SUMIGRAPH NC900, Shimadzu). Exchangeable cations (Ca^2+^, Mg^2+^, K^+^ and Na^+^) were extracted by 1 M ammonium acetate (pH 7.0) according to Schollenberger methods (Schollenberger and Simon 1945) and assayed in solution by flame atomic absorption spectrometry (Z-2310, Hitachi). Cation exchange capacity (CEC) was measured according to semimicro-Schollenberger method (Committee of Soil Environmental Analysis 1970) using 1 M potassium chloride solutions. Available phosphate (available P) was determined using Truog’s method (Truog 1930). Available P was extracted for 30 min using 0.001 M sulphuric acid solution buffered with ammonium sulphate (pH 3), and 8 mL colour-producing reagent (100 mL of 2.5 M sulphuric acid mixed with 30 mL of ammonium molybdate solution, 60 mL ascorbic acid solution and 10 mL of tartar emetic solution) was added. After 18 min, available P was measured with an ultraviolet and visible spectrophotometer (V-600, Jasco). The peak wavelength was 710 nm. Soil nitrate nitrogen (${\text{NO}}_{3}^{ - }$-N) was extracted with water (1:5 soil–water suspensions) for 30 min and quantified using ion chromatography (HIC-SP, Shimadzu). The phosphate absorption coefficient (P absorption) was determined according to Methods of soil environmental analysis (Committee of Soil Environmental Analysis 1970). P absorption was measured with an ultraviolet and visible spectrophotometer (V-600, Jasco). The peak wavelength was 660 nm. Acid oxalate-extractable Al, Fe and Si (Al_o_, Fe_o_ and Si_o_) were determined according to Blakemore et al. (1981). Al_o_, Fe_o_ and Si_o_ were measured with a plasma emission spectrometer (Optima 7300DV, PerkinElmer).

### Preparation and description of soil thin sections

Thin soil sections were prepared according to Nagatsuka and Tamura (1986). Soil core samples were freeze-dried and impregnated with a resin [mixed polyester resin A:polyester resin B (Maruto) = 8:2 1000 mL and benzoyl peroxide 10 mL]. Hardened samples were cut to smaller pieces of approximately 50 × 50 × 7 mm^3^ using a cutting machine (MC-32, Maruto) to obtain thin vertical sections of soil. The samples were ground using an abrasive (C3000, Maruto) (first polishing) and bonded onto a glass slide with an epoxy resin. Samples were cut again and ground to a thickness of 30 µm using an automatic polishing machine, followed by manual polishing with an abrasive (C400 and C3000, Maruto) (second polishing). We described the soil micromorphology according to the Handbook for Soil Thin Section Description (Bullock et al. 1985) based on observations using a polarizing microscope (BH-2, Olympus). The size threshold between coarse and fine materials (the C/F concept) was 10 µm, and the C/F concept and abundance of voids were determined under plane-polarized light.

### Image analysis and measurement of fractal dimension

Optical microscopy images of soil thin sections were used for image analysis. BMP images (698 × 525 pixels) were acquired using an object magnification of 4×, which provided a resolution of 9.5 µm pixel^−1^ in Eniwa and 5.0 µm pixel^−1^ in Narita. The colour microscopy images were converted into monochrome images with image analysis software (A-zôkun, Asahi Kasei Engineering Corporation). The void ratio (%) was determined using the grading analysis functions in A-zôkun. Fractal dimensions were automatically calculated using the fractal analysis system [fractal 3, National Agriculture and Food Research Organization (NARO)] based on BMP images (Sasaki et al. 1994).

Fractal dimensions were calculated using the box counting method (Dathe et al. 2001; Sasaki et al. 1994; Tamura et al. 1993). The objects were covered by orthogonal line grids with an increasing lattice constant. The number (*N*) of meshes (boxes) that contained any part of the structure was determined for each box size (equal to the lattice constant, ε). According to the macro-scale increase in the box size (ε) at selected step sizes and for each box size, boxes containing at least 1 pixel of the contour line were counted (*N*). This count number *N* (ε) depends on the box size (ε) and fractal dimension *D* according to Eq. (3) (Takayashu 1986).

3 $$N(\varepsilon ) \propto \varepsilon^{ - D}$$

Thus, a double-logarithmic plot yields a straight line for fractal objects,

4 $$\log \,N(\varepsilon ) = - D\,\log \,\varepsilon + a$$

and the fractal dimension *D* can be determined as the absolute value of its slope. The constant *a* denotes the ordinate intercept. The lattice constant ε was increased from 4 to 8, 16, 32, 64, 128 and 256 pixels with the software (Sasaki et al. 1994).

### Analytical methods for determining soil biological properties

Microbial biomass carbon (*B*_*C*_) and microbial biomass nitrogen (*B*_*N*_) were measured using the fumigation extraction method (Joergensen and Brookes 1990; Vance et al. 1987). *E*_*C*_ (difference in the total organic carbon content between fumigated and non-fumigated soils) was determined using a total organic carbon analyser (TOC-5000A, Shimadzu) and B_*C*_ was calculated by Eq. (5).

5 $$B_{C} = 2.64 \times E_{C}$$

*B*_*N*_ was measured with the ninhydrin-reactive nitrogen measurement method (Joergensen and Brookes 1990). *B*_*N*_ was calculated by Eq. (6):

6 $$B_{N} = 5.0 \times E_{NIN}$$

where *E*_*NIN*_ is the difference between fumigated and non-fumigated soils in the extracted concentration of ninhydrin-reactive solution.

β-Glucosidase activity was determined according to Hayano (1973) and protease activity was determined according to Ladd and Butler (1972). Each soil biological analysis was replicated three times.

### Effects of soil characteristics on crop productivity

To determine effective soil characteristics associated with crop productivity of UFs, we used a weighted-means approach (Rhoton and Lindbo 1997), whereby individual soil physical, chemical and biological properties were compared among horizons with various management profiles using Eq. (7):

7 $${\text{Mw}} = \Sigma (T \cdot I)/\Sigma T$$

where Mw is the weighted mean, *T* is the thickness of the horizon in cm and *I* is the value of a soil parameter. The thickness of the profile was 75 cm for the physical parameters, 100 cm for the chemical parameters and 20 cm for the biological parameters. We calculated the weighted mean values of physical [bulk density, P_mi_, P_ma_, total pores (P_mi_ + P_ma_), SHC, the thickness of effective soil depth (ESD) and fractal dimension], chemical (OC, TN, exchangeable cations, CEC, base-saturation, ${\text{NO}}_{3}^{ - }$-N and available P) and biological (*B*_*C*_, *B*_*N*_, β-glucosidase and protease) parameters to analyse the correlation between dry yield and each soil parameter. The relationship between dry yield and each soil parameter was evaluated using Pearson’s correlation coefficient (n = 4).

To evaluate the effect of ESD on productivity, we calculated total amounts of plant nutrients in ESD of four farms and compared them between UFs and CFs in Eniwa and Narita.

### Statistical analysis

All of the parameters were tested using an *F* test (two-tailed test, at *p* < 0.05), and the separate means were compared using Student’s *t* test (n = 3, two-tailed test). Student’s *t* test was performed at significance levels of *p* < 0.05, 0.01 and 0.001. The fractal dimensions were tested using ANCOVA.

## Results and discussion

### Crop yield

The mean dry yield of E-UF was 3.2 t ha^−1^ crop^−1^ (tomato) and that of N-UF was 4.2 t ha^−1^ crop^−1^ (green onion) (Table 2). These yields were higher than those of CFs (E-CF: 2.6 t ha^−1^ crop^−1^; N-CF: 2.0 t ha^−1^ crop^−1^) (Table 2). The average unit dry yield of tomato in Japan from 2010 to 2014 is 2.2 t ha^−1^ crop^−1^ and that of green onion is 2.0 t ha^−1^ crop^−1^ [calculated from the average unit yields of tomato (36.2 t ha^−1^) and green onion (20.9 t ha^−1^) and moisture of tomato (94.0 %) and green onion (90.6 %)] (MAFF 2015; Ishiyaku Publishers 2011). Those are the standard conventional yield values for Japan. Thus, the mean dry yields of the studied UFs were higher than the average unit yields in Japan.

**Table 2:** Mean dry yield on Eniwa and Narita farms

Sample	Mean dry yield (t ha^−1^ crop^−1^)	Plants
Eniwa unfertilised farm	3.2	Tomato
Eniwa control farm	2.6	Tomato
Narita unfertilised farm	4.2	Green onion
Narita control farm	2.0	Green onion

### Soil profile morphology and physical properties

The compactness values of 2A of E-CF and A1 of N-CF were 0.718 and 1.636 MPa higher than those of other horizons, whereas all of the horizons of E-UF and N-UF had approximately the same compactness values (Table 3). Miyoshi (1972) reported that a high compactness value over 0.838 MPa imposes a limitation on fine root growth. Therefore, the A1 horizon of N-CF imposed a limitation on the fine root growth. The solid-phase ratio and bulk density of core samples from the Ap3 horizon of E-CF and A1 horizon of N-CF were significantly higher than the A2 horizon of E-UF and A1 horizon of N-UF, respectively (Table 4). The pore spaces and SHC in the Ap3 and A1 horizons of E-CF and N-CF, respectively, were significantly lower than those in the A2 and A1 horizons of E-UF and N-UF, respectively (Table 4). In farmland, pressure pans are generally highly compacted, with a high bulk density and low porosity immediately below the ploughed layer due to pressure from the tractor (Morph and Tech 2006; Kato 2014). Pagliai et al. (2004) studied porosity and soil thin sections under different tillage management regimes, which were (1) harrowing with a disc harrow to a depth of 10 cm (minimum tillage) and (2) mouldboard ploughing to a depth of 40 cm (conventional deep tillage). They showed that micro porosity within aggregates under minimum tillage was higher than that under conventional deep tillage. They also reported that conventional deep tillage yielded low porosity in a layer of 40–50 cm depth, which showed a plough pan in a soil thin section. Ciarkowska (2010), studying the effect of fertilisation on soil structure, reported that microstructure in the 0–10 cm horizon of chemically fertilised soil was weakly developed and that the soil microstructure had lower porosity than that of manured or unfertilised soils. In this study, the Ap3 horizon of E-CF and the A1 horizon of N-CF contained a pressure pan under the ploughed layer, whereas both UFs lacked pressure pans. This result suggested that the pressure pan of E-CF was caused by deep tillage management. In N-CF, the formation of a pressure pan may have been affected by application of chemical fertilisers.

**Table 3:** Profile descriptions for the four soils on Eniwa and Narita farms

Horizon	Depth (cm)	Colour	Texture (USDA)	Rock fragments^a^	Structure^b^	Compactness^c^ (MPa)	Roots^d^
E-UF							
Oi	+1–0						
Ap	0–9	10YR2/1	SL	W/C	3/M/Cr	0.096	Vf/M, F/Vf
A1	9–25	10YR2/1	SL	W/C	3/F/Cr, 2/M/Sb	0.137	Vf/M, F/Vf
A2	25–40	10YR2/1	SL	W/C	3/M/Cr, 2/M–C/Sb	0.296	Vf/M, F/Vf, M/F
2A3	40–55	10YR1.7/1	CL	None	2/F/Sb	0.396	VF/C, F/F
3AB	55–75	10YR2/2	CL	ST/M	2/F–C/Sb	0.617	Vf/F, F/Vf
4Bw	75–100+	10YR4/6	L	ST/A	2/F–C/Sb	1.156	None
E-CF							
Ap1	0–12	10YR2/1	SL	W/C	3/F–C/Cr	0.161	Vf/C, F/Vf
Ap2	12–30	10YR2/1	SL	W/C	2/F–C/Cr	0.116	Vf/C, F/F, M/Vf
Ap3	30–40	10YR2/1	SL	W/A	2/F–C/Cr, 1/M–C/Sb	0.342	Vf/F, F/Vf
2A	40–50	10YR1.7/1	CL	ST/F	2/M–C/Sb	0.718	Vf/Vf
3AB	50–70	10YR3/4	CL	ST/M	2/M–C/Sb	0.532	Vf/Vf
4Bw	70–100+	10YR4/6	SL	ST/A	1/C/Sb	1.370	None
N-UF							
Ap	0–8	7.5YR2/3	SIC	None	3/F/Cr, 1/M/Sb	0.038	Vf/M, F/M
A1	8–21	7.5YR2/2	C	None	2/F–C/Sb	0.459	Vf/M, F/M
A2	21–45	7.5YR2/2	C	None	2/F–C/Sb	0.459	Vf/F, F/F
AB	45–61	7.5YR3/2	SIC	None	2/F–C/Sb	0.396	Vf/F, F/F
Bw1	61–87	7.5YR4/4	SIC	None	2/F–C/Sb	0.459	Vf/F, F/F
Bw2	87–100+	7.5YR4/6	SIC	None	2/F–C/Sb	0.718	Vf/F, F/F
N-CF							
Ap	0–17	7.5YR2/2	C	None	3/M/Cr	0.050	Vf/F, F/F
A1	17–30	7.5YR2/2	C	None	2/F–C/Sb	1.636	Vf/VF
A2	30–44	7.5YR3/2	SIC	None	2/F–C/Sb	0.396	Vf/F, F/F
AB	44–62	7.5YR4/4	SIC	None	2/F–C/Sb	0.617	Vf/VF, F/Vf
Bw	62–100+	7.5YR4/6	SIC	None	2/F–C/Sb	0.718	Vf/Vf, F/Vf

Profiles are described according to a soil description system of United Soil Classification System of Japan (The Fourth Committee for Soil Classification and Nomenclature in the Japanese Society of Pedology 2010)

^a^State of wethering (*W* weathered, *ST* strongly weathered)/abundance (*F* few, *C* common, *M* many, *A* abundant), all rock fragments were gravel or stone size and shape ware rounded

^b^Grade (1: weak, 2: moderate, 3: strong)/size (*F* fine, *M* medium, *C* course)/type (*Cr* crumb, *Sb* subangular blocky)

^c^Compactness was measured by using soil hardness tester (DIK-5553, Daiki)

^d^Size (*Vf* very fine, *F* fine, *M* medium)/abundance (*Vf* very few, *F* few, *C* common, *M* many)

**Table 4:** Physical properties of the soils sampled from Eniwa and Narita farms

Horizon	Depth (cm)	Three phase distribution (%)			Porosity (%)		Bulk density^a^ (Mg m^−3^)	SHC^b^ (K_20_ cm^−1^)
		Solid	Liquid	Gaseous	P_ma_	P_mi_		
E-UF								
Ap	0–5	31.4	34.0	34.6	15.1	53.5	0.85 ± 0.03	3.9 × 10^−2^
A1	10–15	30.4	31.0	38.6	19.2	50.4	0.84 ± 0.02	8.3 × 10^−2^
A2	30–35	29.8*	31.9	38.3	19.1*	51.1	0.83 ± 0.02*	4.9 × 10^−2^*
2A3	50–55	13.1	54.6	32.3	13.6	73.3	0.37 ± 0.03*	3.1 × 10^−2^
3AB	70–75	16.6	61.4	22.1	11.6	71.8	0.45 ± 0.01**	6.0 × 10^−3^
E-CF								
Ap1	0–5	32.7	33.5	33.7	14.5	52.7	0.90 ± 0.03	5.7 × 10^−2^
Ap2	12–17	29.9	31.9	38.2	18.4	51.7	0.84 ± 0.02	4.7 × 10^−2^
Ap3	30–35	33.0*	37.1	29.9	12.9*	54.1	0.92 ± 0.03*	1.6 × 10^−2^*
3AB	50–55	14.0	61.9	24.1	11.2	74.8	0.43 ± 0.01*	1.9 × 10^−3^
4Bw	70–75	20.7	59.8	19.4	10.5	68.7	0.55 ± 0.03**	3.1 × 10^−3^
N-UF								
Ap	0–5	27.8	18.8	53.4	17.4	54.8	0.72 ± 0.07	2.24 × 10^−2^
A1	10–15	29.7*	29.3	41.0	13.4	56.9	0.80 ± 0.10**	3.15 × 10^−2^
A2	30–35	23.7	48.1	28.3	10.7	65.7	0.70 ± 0.01**	6.19 × 10^−4^**
AB	50–55	18.0	53.5	28.5	11.7	70.3	0.55 ± 0.04	1.15 × 10^−3^
Bw1	70–75	16.8**	58.6	24.6	11.5	71.8	0.51 ± 0.004**	5.78 × 10^−4^
N-CF								
Ap	0–5	24.1	28.9	47.0	23.9	52.0	0.69 ± 0.02	2.29 × 10^−2^
A1	20–25	41.1*	42.0	16.9	8.5	50.4	1.09 ± 0.02**	1.28 × 10^−3^
A2	35–40	21.8	43.9	34.3	13.8	64.4	0.62 ± 0.02**	2.98 × 10^−3^**
AB	50–55	16.9	49.2	34.0	14.0	69.2	0.51 ± 0.01	1.94 × 10^−3^
Bw	70–75	15.1**	52.2	32.7	12.6	72.3	0.47 ± 0.01**	2.71 × 10^−3^

*, ** Significant at the 0.05 and 0.01 probability levels among depths between UF and CF at the same location, respectively

^a^Bulk density was shown as average ± SD

^b^Saturated hydraulic conductivity. Water temperature was 20 °C

In crop production, it is important that ‘the effective soil depth (ESD)’ which has a low compactness value (<0.718–0.982 MPa) because the growth of fine roots is restricted with higher compactness (Fuziwara et al. 2010; Saigusa 2014). In the present study, ESD values were 87, 75, 40 and 17 cm in N-UF, E-UF, E-CF and N-CF, respectively. Thus, ESDs of UFs were thicker than those of CFs.

The soils on the farms evaluated in this study were composed of andosols (Table 1), which develop in volcanic areas and contain a high proportion of glass and short-range-order materials, including allophane and imogolite (IUSS Working Group 2014; Shoji et al. 1993). Andosols provide favourable condition for cultivation, plant roots and water storage because they have a lower bulk density and a higher amount of organic matter than another soil types (IUSS Working Group 2014). Karasawa et al. (2015) reported that crop yields of organic farms were lower than those of conventional farms in the first year during the organic transition period; however, yields increased to equal those of conventional farms after 3 years in an andosol. This suggests that andosol soils have relatively thicker layers than other soil types and can change physical properties during the several years.

### Soil chemical properties

The soil pH was mildly acidic in all profiles from Eniwa and Narita, at 5.43–6.45 (Table 5). Among the Ap horizons in all profiles, the highest OC concentration was E-UF, indicating that weed mulch (Oi horizon) affected OC in the surface soil of E-UF (Table 5). The concentrations of OC and TN in the AB horizons of UFs were higher than those of CFs. The CEC was significantly correlated with OC [r = 0.986*** in Eniwa (n = 12) and r = 0.844*** in Narita (n = 11)]. CEC is dependent on electrically charged surfaces of the soil colloidal fraction consisting of soil organic matter or clay minerals (Brady and Weil 2008). Organic carbon input into subsoils occurs in dissolved form (DOC) following preferential flow pathways, as aboveground or root litter and exudates along root channels and/or through bioturbation (Rumpel and Kögel-Knabner 2011). In this study, many fine roots were observed in the B horizon of UFs (Table 3), suggesting that the increased of CEC was due to root system activity. The concentrations of exchangeable cations (Ca^2+^, Mg^2+^ and K^+^) in the surface horizons of CFs tended to be higher than those in the surface horizons of UFs. The base-saturation percentage exhibited the same trend as the exchangeable cations. The concentration of ${\text{NO}}_{3}^{ - }$-N tended to increase continuously with the horizon depth of E-UF, N-UF and N-CF, and the lowest concentration were found in E-CF. In Narita, the concentration of ${\text{NO}}_{3}^{ - }$-N was higher in the Ap horizon of UF than in that of CF; however, the concentration of ${\text{NO}}_{3}^{ - }$-N in the subsoil of CF was higher in the subsoil of UF. In the upper horizons of the Eniwa farms (Ap–A2 of E-UF and Ap1–Ap3 of E-CF), the P absorption values were lower than 1500 g 100 g^−1^ (Table 5). Available P concentrations in the surface horizons were lower in E-UF than in E-CF (Table 5), suggesting that the low concentration of available P in E-UF was because of unfertilised management. In contrast, on the Narita farms, the P absorption values were higher than 1500 g 100 g^−1^ in all horizons (Table 5). Available P was in the same concentration in both N-UF and N-CF (Table 5). These results suggest that phosphorus derived from fertilisers was adsorbed to the soil in N-CF because phosphorus fixation of soil was strong in Narita farm. The pH (NaF) from Eniwa and Narita ranged from 9.85 to 12.01 (Table 6). In Eniwa, there was no difference in Al_o_ between UF and CF. In contrast, in the surface horizon of N-UF, the values of pH(NaF) and Al_o_ tended to be lower than that of N-CF (Table 6). In andosols, aluminium ions derived from weathered volcanic ash bind humic substances as an organo-mineral complex (Shoji et al. 1993). Thus, the results suggested that more aluminium ions were bound to humic substance in the surface horizon of N-UF than in that of N-CF.

**Table 5:** Chemical properties of the soils sampled from Eniwa and Narita farms

Horizon	pH		OC	TN	Exchangeable cation (cmol_c_ kg^−1^)				CEC (cmol_c_ kg^−1^)	Base-satu. (%)	${\text{NO}}_{3}^{ - }$ (mg kg^−1^)	Available P (mg kg^−1^)	P absorption (mg 100 g^−1^)
	H_2_O	KCl	(g kg^−1^)		Ca^2+^	Mg^2+^	K^+^	Na^+^					
E-UF													
Ap	6.2	5.07	55.2	3.4	9.78	0.93	0.41	0.04	17.8	62.7	9.88	112.0	970
A1	6.1	5.05	56.1	3.4	10.01	0.94	0.21	0.06	17.3	64.9	9.25	119.6	1170
A2	6.0	4.96	53.9	3.2	9.53	0.84	0.14	0.06	18.6	57.0	9.39	84.1	1314
2A3	5.4	4.48	168.8	9.4	7.14	0.30	0.33	0.17	51.5	15.4	23.36	n.d.	2924
3AB	5.6	4.93	74.5	4.8	3.36	0.23	0.38	0.08	27.3	14.9	15.35	n.d.	2707
4Bw	5.7	5.40	20.3	1.5	2.27	0.23	0.09	0.12	11.0	24.7	15.16	n.d.	2123
E-CF													
Ap1	6.5	5.30	44.8	2.7	12.54	0.94	0.45	0.05	18.7	74.7	4.10	233.1	1223
Ap2	6.3	5.27	40.5	2.5	14.05	1.45	0.28	0.05	20.2	78.2	5.80	293.2	1282
Ap3	6.4	5.37	54.1	3.1	13.94	1.25	0.24	0.05	19.8	78.2	3.55	270.8	1386
2A	5.6	4.66	163.2	9.1	10.43	0.72	0.35	0.09	45.6	25.4	4.36	n.d.	2876
3AB	5.7	5.23	39.6	2.8	2.42	0.24	0.35	0.06	17.5	17.5	3.21	n.d.	2570
4Bw	5.7	5.49	11.9	0.9	1.38	0.11	0.23	0.07	7.1	25.4	3.43	n.d.	1729
N-UF													
Ap	5.6	4.32	38.0	3.0	8.89	2.85	0.74	0.13	28.7	44.0	15.08	244.6	1561
A1	5.6	4.28	35.2	2.7	8.24	2.84	0.40	0.15	28.4	40.9	3.35	77.1	1522
A2	6.0	4.74	30.9	2.4	13.17	3.14	0.28	0.49	30.3	56.3	2.80	n.d.	2052
AB	5.9	4.84	38.4	2.8	10.01	2.10	0.12	0.41	30.6	41.3	3.46	n.d.	2466
Bw1	5.9	5.37	28.5	2.5	7.53	1.38	0.13	0.19	24.8	37.2	10.13	n.d.	2723
Bw2	6.2	5.66	15.7	1.4	6.99	1.62	0.14	0.20	22.0	40.7	10.56	n.d.	2939
N-CF													
Ap	6.4	5.09	43.0	3.3	15.02	3.71	1.79	0.09	35.4	58.3	9.06	201.9	1572
A1	6.1	4.76	34.1	2.5	11.37	2.81	1.57	0.09	23.2	68.3	3.57	68.1	1614
A2	5.6	4.82	41.4	2.7	12.80	2.88	1.67	0.19	34.4	51.0	3.70	n.d.	2147
AB	5.9	5.12	34.6	2.6	6.00	2.21	0.25	0.24	29.1	29.9	13.44	n.d.	2518
Bw	5.9	5.45	22.6	2.0	10.46	1.31	0.11	0.13	21.2	56.6	21.40	n.d.	2681

*n.d.* not detected

**Table 6:** pH(NaF) and acid oxalate-extractable Al, Fe and Si of the soils sampled from Eniwa and Narita farms

Horizon	pH	Acid-oxalate (g kg^−1^)			Al_o_ + 1/2Fe_o_ (%)
	NaF	Al_o_	Fe_o_	Si_o_	
E-UF					
Ap	11.96	12.11	6.10	2.72	1.52
A1	11.98	10.50	5.32	2.31	1.32
A2	11.98	12.44	5.79	2.76	1.53
2A3	10.27	58.22	16.47	15.96	6.65
3AB	11.28	70.53	17.92	27.33	7.95
4Bw	11.72	56.86	9.78	27.41	6.18
E-CF					
Ap1	12.01	10.87	5.74	2.41	1.37
Ap2	11.97	11.91	6.15	2.74	1.50
Ap3	11.98	13.02	6.12	2.99	1.61
2A	10.57	57.42	17.38	15.55	6.61
3AB	11.55	71.11	15.82	29.90	7.90
4Bw	11.92	46.40	6.35	22.04	4.96
N-UF					
Ap	9.85	13.74	18.16	4.42	2.28
A1	9.94	13.41	17.73	4.60	2.23
A2	10.35	15.83	23.42	6.62	2.75
AB	10.96	31.67	29.65	14.63	4.65
Bw1	11.21	54.36	34.18	30.06	7.14
Bw2	11.23	98.90	37.09	59.73	11.74
N-CF					
Ap	10.31	18.10	19.18	7.11	2.77
A1	10.36	18.30	19.70	7.49	2.81
A2	10.72	25.79	28.30	11.44	3.99
AB	11.13	49.38	34.99	25.54	6.69
Bw	11.23	63.34	39.26	35.85	8.30

### Soil micromorphology

The common characteristics of the Eniwa farms were a chitonic and enaulic c/f-related distribution, including large amounts of pumice (with white colour) as coarse fragments and dark fine particles in the upper layer, whereas a porphyric c/f-related distribution dominated in the lower layers, with dark fine particles in the buried A horizons and yellowish pumice fragments in the Bw horizons. At Narita, all the horizons had monic c/f-related distributions of fine particles. In terms of a soil structure, there were no differences in the Ap (0–5 cm) horizons of the four profiles but there were differences in some horizons below the Ap horizon of UFs and CFs. The four profiles were defined based on the micromorphological descriptions given below.

**Fig. 1:**
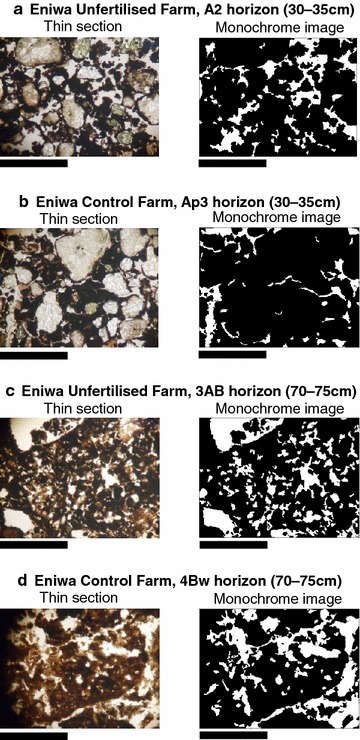
Soil thin sections and monochrome images of the soil microstructure on Eniwa farms. *Bar* 3.0 mm. **a** Well-developed granular structure in the A2 horizon of E-UF. Granules include pumice stone and large amounts of excrements. **b** Subangular blocky structure in the Ap3 horizon of E-CF. **c** Spongy structure with channels in the 3AB horizon of E-UF. **d** Subangular blocky structure with strongly weathered pumice stone in the 4Bw horizon of E-CF

**Fig. 2:**
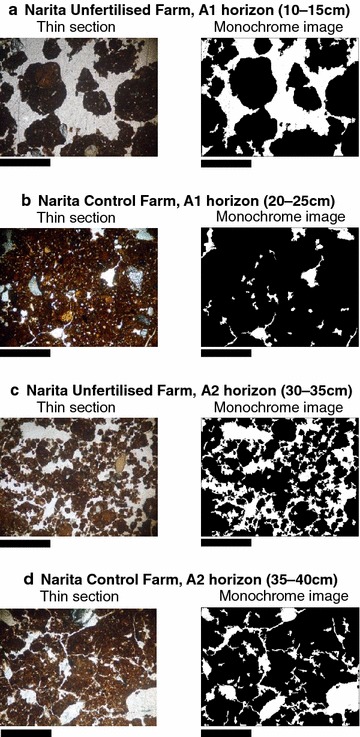
Soil thin sections and monochrome images of the soil microstructure at the Narita farms. *Bar* 1.0 mm. **a** Well-developed granular structure in the A1 horizon of N-UF. **b** Weakly developed granular structure in the A1 horizon of N-CF. The granules have been compressed. **c** Spongy structure with crumbs in the A2 horizon of N-UF. This structure contains many channels. **d** Subangular blocky structure with planes and channels in the A2 horizon of N-CF

The pressure pan horizons were dominated by a subangular blocky structure and a weakly developed granular structure with planes in the Ap3 (30–35 cm) horizon of E-CF (Table 7; Fig. 1b) and in the A1 (20–25 cm) horizon of N-CF (Table 7; Fig. 2b). The granules in these structures were consolidated and had a low (7–11 %) void ratio (Table 7). Thus, these horizons appeared to have low permeability (Table 4). In contrast, the soil microstructures of UFs were dominated by a well-developed granular structure in the upper layer [the A1 (10–15 cm) and A2 (30–35 cm) horizons in E-UF and the A1 (10–15 cm) horizon of N-UF (Table 7; Figs. 1a, 2a)] compared with the pressure pan horizons of CFs. Additionally, a spongy structure constructed mainly of crumbs and granules was present in the lower horizons of UFs [the 2A3 (50–55 cm) and 3AB (70–75 cm) horizons in E-UF and the A2 (30–35 cm) horizon in N-UF (Table 7; Figs. 1c, 2c)]. This structure had a high (19–33 %) void ratio (Table 7). A similar pattern has been observed in the surface horizons of organic farms (Gerhardt 1997; Pulleman et al. 2003; Papadopoulos et al. 2014). However, in subsoil, subangular blocky and/or blocky microstructures were observed in the B horizon (40 cm) of organic farms that were managed for 2 years (Gerhardt 1997). In contrast with previous studies, our results clearly demonstrated that a spongy structure developed in the subsoil horizons of UFs that were managed for over 40 years.

**Table 7:** Micromorphology of the soils thin sections from Eniwa and Narita farms

Horizon and depth (cm)	Dominant microstructure^a^	Grade of pedality^b^	Size of peds (mm)	Voids		Fractal dimension of voids^d^	Basic organic components	Pedofeatures
				Types^c^	Abundance (%)			
E-UF								
Ap (0–5)	Gr and Igm	M–S	0.5–4.0	Cdp	19	1.556*	Large amount of root tissues and fungal hyphae in Ap–A2, and some root tissues and fungal hyphae in 2A3 and 3AB	Large amount of excrements in Ap–A2
A1 (10–15)	Gr	S	0.3–5.0	Cdp	24	1.640		
A2 (30–35)	Gr	M–S	0.5–5.0	Cdp	19	1.580**		
2A3 (50–55)	Sp	M	0.1–2.5	Chn, Vu	20	1.572*		
3AB (70–75)	Sp	M	0.1–2.2	Chn, Vu	27	1.631		
E-CF								
Ap1 (0–5)	Gr	M–S	0.2–5.0	Cdp	13	1.485*	Some root tissues and fungal hyphae in Ap1–Ap3	Some excrements in Ap1–Ap2
Ap2 (12–17)	Gr	S	0.5–8.0	Cdp	25	1.631		
Ap3 (30–35)	Sb	M	1.5–10.0	Cdp, Pn	11	1.446**		
3AB (50–55)	Sp	W	0.1–1.3	Vu	14	1.506*		
4Bw (70–75)	Sb	W–M	0.3–3.0	Vu, Pn	29	1.654		
N-UF								
Ap (0–5)	Gr	S	0.3–5.0	Cdp	32	1.685	Some root tissues, carbide and fungal hyphae in Ap–AB	Very few amorphous nodules in Ap–A1
A1 (10–15)	Gr	S	0.3–5.0	Cdp	33	1.692***		
A2 (30–35)	Sp	M–S	0.1–3.0	Cdp, Chn	33	1.681***		
AB (50–55)	Sp and Sb	M	0.2–2.5	Chn, Vu, Pn	13	1.484*		
Bw1 (70–75)	Sp and Sb	M	0.3–4.0	Chn, Vu, Pn	16	1.518		
N-CF								
Ap (0–5)	Gr	S	0.3–4.0	Cdp	23	1.638	Few tissues, carbide and fungal hyphae in Ap–A2	Very few amorphous nodules in A1
A1 (20–25)	Gr	W	0.3–5.0	Cdp, Pn	7	1.366***		
A2 (35–40)	Sb	M–S	0.5–2.5	Cdp, Pn, Chn	22	1.589***		
AB (50–55)	Sp and Sb	M	0.2–3.0	Chn, Vu, Pn	17	1.552*		
Bw (70–75)	Sp and Sb	M	0.1–3.0	Chn, Vu, Pn	18	1.553		

^a^Microstructure (*Gr* granular structure, *Igm* intergrain microaggregate structure, *Sp* spongy structure, *Sb* subangular blocky structure)

^b^Grade of pedality (*S* strongly developed, *M* moderately developed, *W* weakly developed)

^c^Types of voids (*Cdp* compound packing, *Chn* channels, *Vu* vughs, *Pn* planes)

^d^Fractal dimension of voids [*, **, ***: significant at the 0.05, 0.01 and 0.001 probability levels among depths between UF and CF at the same location, respectively (ANCOVA)]

In all horizons, there was a strong linear relationship between log_10_[N(ε)] and log_10_(ε), according to Eq. (4), which indicated that the microstructures studied had fractal properties, and thus, fractal dimensions could be calculated. The fractal dimension values for Ap, A2 and 2A3 of E-UF and A1 and A2 of N-UF were higher than those of E-CF and N-CF, respectively (Table 7). High fractal dimension values indicate greater soil structure complexity (Tamura et al. 1993); thus, the soil structure of UFs was of higher complexity than that of CFs. Furthermore, the spongy microstructure demonstrated a high fractal dimension in addition to a well-developed granular microstructure.

**Fig. 3:**
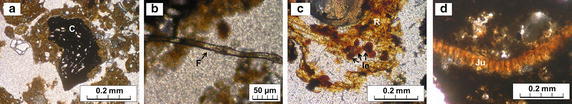
Basic organic components and pedofeatures in the soil thin sections. **a** Plant charcoal (C) from the Ap horizon of N-UF. **b** Fungal filaments (F) from the Ap horizon of N-UF. **c** Intact excrement pedofeature (Ie) in a root residue (R) from the A1 horizon of E-UF. **d** Fauna such as Julidae (Ju) from the Ap horizon of E-UF

The basic organic components comprised many plant root organs and tissue residues, cell residues of plant roots, organic pigments, yellow fungal filaments (50–300 µm in diameter) and colourless fungal filaments (10 µm in diameter) in the upper part of the profile of E-UF (Table 7). The upper horizons of N-UF and N-CF also contained plant charcoal (Fig. 3a), plant roots, stem residues (organs) and dark brownish amorphous organic fine materials infiltrating the micromass. The Ap and A1 horizons of N-UF and the Ap horizon of N-CF contained fungal filaments (with no clear colour and 10 µm in diameter) (Fig. 3b). Among the pedofeatures in the Ap–A2 horizons in E-UF, red or brown intact excrement (Fig. 3c) formed egg-shaped aggregates measuring approximately 0.03–0.2 mm in diameter with smooth surfaces, which were distributed in the plant residue, and large amounts of black aged excrement formed very porous micro-aggregates in the matrix. In addition, fauna such as Julidae were observed in the Ap horizon of E-UF (Fig. 3d). The amount of organic components and excrements in the upper horizons of UFs exceeded that of CFs (Table 7).

The aggregate development and stability of soils is influenced by root systems, fungal filaments and soil fauna excrement (Daynes et al. 2013; Oades 1993; Ritz and Young 2004; Tisdall and Oades 1982). According to the soil thin sections, the percentage of organic components (root residues and hyphae) and excrement pedofeatures was higher in UFs than in CFs (Table 7). Thus, we propose that the formation of the well-developed granular microstructure of surface soil and spongy microstructure of subsoil was affected by plant roots, soil fauna and soil microorganisms, particularly fungal hyphae. We also detected differences between the upper and lower layers of UFs with respect to the structures observed. A granular structure is common in surface horizons with a very fine silt to clayey texture (such as in vertisols) (Fitzpatrick 1984; Kovda and Mermut 2010) or in mollic subsurface horizons with many grass roots (Gerasimova and Lebedeva-Verba 2010; Oades 1993). Shrink–swell processes in clay with a dry–wet cycle and the presence of root systems lead to the development of granular aggregates in these soils (Oades 1993). In the present study, the granular microstructures of the A1 and A2 horizons of E-UF and the A1 horizon of N-UF contained many root residues and tissues (Table 7), and thus, the development of the granular microstructure was affected by root systems. In general, volcanic ash soils have a small granular microstructure (Sedov et al. 2010). In both Eniwa farms, the buried horizons were dominated by spongy microstructures composed of moderately or strongly developed granules, suggesting that this structure was influenced by volcanic ash. However, on the Narita farms, the spongy structure was found only in the A1 horizon of N-UF and not that of N-CF. The aggregate hierarchy concept, different binding agents act at different hierarchical stages in soil aggregates (Tisdall and Oades 1982). Macro-aggregates (0.25–5 mm in diameter) comprise many micro-aggregates (2–250 µm), which are bound together mainly by a sticky network formed from fungal hyphae and fine roots. The micro-aggregates comprise mainly fine sand grains and small clumps of silt grains, clay and organic debris, which are bound together by root hairs, fungal hyphae and microbial gums (Brady and Weil 2008; Six et al. 2004; Tisdall and Oades 1982). In the soil thin sections, the granules in the A1 horizon of N-UF measured 0.3–5.0 mm in diameter, whereas the small granules in the crumbs in the A2 horizon of N-UF measured approximately 100–300 µm in diameter. These results show that the granular structure was dominated by macro-aggregates, whereas the spongy structure was dominated by micro-aggregates. The difference of aggregate size between the granular and spongy structures suggested that different organic matter act as binding agents with the granular structure affected by comparatively large roots and hyphae and the spongy structure affected by root hairs, fine fungal hyphae and microbial debris.

### Soil biological properties

*B*_*C*_ levels in the Ap horizons of E-UF and N-UF and the A1 horizon of N-UF were significantly higher than those of E-CF and N-CF, respectively (Table 8). In the Ap horizon of the Eniwa farms, *B*_*N*_ level and β-glucosidase activity were significantly higher in E-UF than that in E-CF (Table 8). The protease activity levels in the Ap and A1 horizons of E-UF and N-UF were significantly higher than those in the Ap1 or Ap and Ap2 or A1 horizons of CFs (Table 8). Sakurai et al. (2007) reported that the protease activity levels were higher in a site with organic management compared with those in a site with inorganic fertiliser management and that the proteolytic bacterial communities were different under organic management than under inorganic fertiliser management. Our results suggested that the microbial biomass increased and that the microbial communities were changed by unfertilised management.

**Table 8:** Biological properties of the soils sampled from Eniwa and Narita farms

Horizon	*B*_*C*_ (µg g^**−**1^)	*B*_*N*_ (µg g^−1^)	β-Glucosidase (µmol h^−1^ g^−1^)	Protease (µmol h^−1^ g^−1^)
E-UF				
Ap	360.77 ± 19.15**	11.97 ± 1.24**	0.934 ± 0.12**	0.414 ± 0.04**
A1	289.68 ± 33.98	8.37 ± 0.90	0.615 ± 0.07	0.284 ± 0.02**
E-CF				
Ap1	271.75 ± 43.02**	6.76 ± 2.45**	0.512 ± 0.08**	0.102 ± 0.05**
Ap2	250.19 ± 89.74	8.54 ± 2.29	1.194 ± 0.37	0.482 ± 0.07**
N-UF				
Ap	247.51 ± 35.00*	11.26 ± 2.52	1.244 ± 0.24	0.123 ± 0.02**
A1	129.43 ± 3.38*	6.62 ± 3.98	0.685 ± 0.16	0.054 ± 0.02**
N-CF				
Ap	161.07 ± 6.11*	10.24 ± 1.43	0.839 ± 0.04	n.d.
A1	106.48 ± 5.62*	1.74 ± 0.13	0.473 ± 0.10	0.018 ± 0.01**

*n.d.* not detected

*, ** Significant at the 0.05 and 0.01 probability levels among depths between UF and CF at the same location, respectively (Student’s *t* test)

### Effects of soil characteristics on crop productivity of UFs

The weighted mean values of physical, chemical and biological parameters used for analysis of the correlations between dry yield and soil parameters are summarised in Table 9. In the result, the mean dry yield (Table 2) had a positive correlation (p < 0.1) on the thickness of ESD (r = 0.96). Fractal dimension also correlated strongly with dry yield (r = 0.84). The dry yields of UFs were higher than those of CFs (Table 2). Thus, the high productivity of UFs was influenced by the thickness of the ESD and the complexity of the soil structure. First, we considered some influences of ESD and fractal dimension of void on productivity.

**Table 9:** Weighted means of physical, chemical and biological parameters on Eniwa and Narita farms

	Bulk density (Mg m^−3^)	P_ma_ (%)	P_mi_ (%)	Total pores (%)	SHC (cm s^−1^)	ESD (cm)	Fractal dimension	OC (g kg^−1^)	TN (g kg^−1^)
Sample									
E-UF	0.64	15.6	61.2	76.8	4.0 × 10^−2^	75	1.60	67.3	4.1
E-CF	0.74	13.9	59.8	73.7	2.5 × 10^−2^	40	1.53	45.9	2.8
N-UF	0.65	12.2	65.1	77.3	8.4 × 10^−3^	87	1.61	30.6	2.4
N-CF	0.66	15.0	61.7	76.7	6.9 × 10^−3^	17	1.55	32.3	2.5

	Exchangeable cations (cmol_c_ kg^−1^)					CEC (cmol_c_ kg^−1^)	Bse-satu. (%)	${\text{NO}}_{3}^{ - }$-N (mg kg^−1^)	Available P (mg kg^−1^)
	Ca^2+^	Mg^2+^	K^+^	Na^+^	Total				
Sample									
E-UF	6.22	0.51	0.24	0.09	7.07	23.1	36.0	14.14	41.8
E-CF	7.37	0.65	0.30	0.06	8.38	18.0	44.5	4.00	107.8
N-UF	9.41	2.25	0.25	0.29	12.20	27.5	43.9	6.87	29.6
N-CF	10.88	2.29	0.83	0.15	14.15	27.1	52.8	13.07	43.2

	*B*_*C*_ (µg g^−1^)	*B*_*N*_ (µg g^−1^)	β-Glucosidase (µmol h^−1^ g^−1^)	Protease (µmol h^−1^ g^−1^)
Sample				
E-UF	321.67	9.99	0.76	0.342
E-CF	263.12	7.47	0.78	0.254
N-UF	175.28	8.48	0.72	0.082
N-CF	152.88	8.96	0.76	0.003

According to grassland studies, soil depth (associated with topographic position) can affect plant productivity (Briggs and Knapp 1995). Rhoton and Lindbo (1997) reported that a decrease of ESD from 100 to 20 cm caused a decrease in crop productivity and that ESD affected soil quality by determining soil water storage capacity, assuming that nutrient and organic matter levels were generally similar. In this study, we compared total amounts of plant nutrients in ESD of UFs and that of CFs (Table 10). Concentrations of OC, TN and ${\text{NO}}_{3}^{ - }$-N in the soil of UFs were approximately two-times higher than those in CFs (Table 10). Ca^2+^ and Mg^2+^ in ESD were similar in E-UF and E-CF (1114 vs 1185 and 68 vs 65 g m^−2^) and were 1.6 times higher in Narita than in N-CF (1529 vs 944 and 271 vs 167 g m^−2^). K^+^ in E-UF was higher than that in E-CF. Thus, a thick ESD can supply a large amount of nutrients to plant roots under unfertilised conditions. However, available P in ESD of UFs was lower than that of CFs. K^+^ in ESD of N-UF was also lower than that of N-CF. These results suggested that soil P and K tend to decrease under long-term unfertilised conditions. According to Silberbush and Barber (1983), an increase in soil depth by root-length production promotes K uptake in soil. Furthermore, in low-P plots, root length, root turnover and P uptake were increased compared with high-P plots (Lambers et al. 2006). Thus, we considered that large ESD is important for nutrient absorption in plant roots.

**Table 10:** Total chemical amounts in the ESD in Eniwa and Narita farms

	ESD (cm)	OC (kg m^−2^)	TN (kg m^−2^)	Ca^2+^ (g m^−2)^	Mg^2+^ (g m^−2)^	K^+^ (g m^−2^)	${\text{NO}}_{3}^{ - }$-N (g m^−2^)	Available P (g m^−2^)
E-UF	75	34.5	2.08	1114	68	61	11.3	35.1
E-CF	40	15.9	0.95	1185	65	42	5.1	74.9
N-UF	87	18.2	1.42	1529	271	75	3.3	22.1
N-CF	17	9.9	0.74	944	167	203	1.6	33.3

Next, we consider the relationship between fractal dimension and productivity. Soil structure is one of the most important factors that affects crop production because it determines the depth to which roots can penetrate, water retaining capacity and movements of air, water, soil fauna and microorganisms (Hermavan and Cameron 1993; Langmaack 1999; Pagliai et al. 2004). A high fractal dimension value indicates high complexity of the soil structure (Tamura et al. 1993) and high specific surface area (SSA) (Ersahin et al. 2006). The SSA increases CEC as well as the space available for nutrient dynamics and chemical transport processes at the interface between the liquid and solid phases (Ersahin et al. 2006). In addition, an increased SSA provides more available habitat space for microorganisms (Verran and Boyd 2001). Soil microbial habitat segregation occurs among soil aggregate size and pore size distribution (Hattori et al. 1976; Ranjard and Richaume 2001). Bacterial colonies live in the ‘inner part’ of the aggregates in which micropores have a diameter of 2–6 µm. Fungi favour the ‘outer part’ area represented by macro pores with a diameter >6 µm (Hattori et al. 1976; Ranjard and Richaume 2001; Young and Ritz 2000). Thus, in a structure of high fractal dimension, the soil microbial community may become diverse. Several studies of unfertilised or organic experimental plots have shown that the soil microbial biomass is the main nutrient source (such as N and P) for plants (Jenkinson and Ladd 1981). The amounts of TN and phosphorus metabolized by microorganisms were calculated from the metabolic turnover rate (2.5 years), showing that biological metabolic N and P are important sources of plant minerals (Jenkinson and Ladd 1981). According to previous studies regarding unfertilised cultivation systems, biological activity probably influences nutrient cycles for UF crops, with nitrogen fixation being an example (Oda and Hosen 2011; Kuwada et al. 2006).

Thus, this suggests that the granular and spongy structures observed in UF soils increase the sites available for nutrient exchange, for use as habitats by soil microbes and other organisms and for biodiversity of soil microbes and other organisms.

## Conclusion

Our results show that UFs have very thick ESD. Furthermore, we clearly show that the farms under unfertilised conditions featured well-developed soil structures from surface to subsoil. These developed structures had high pore spaces and complexity. ESD and fractal dimension of void had a positive correlation with mean dry yield. Plant root residues, organic pigments and fungal filaments were observed in soil thin section of UFs. Furthermore, in Eniwa, large amount of excrements were observed in surface and subsurface horizon. Organic components and pedofeatures were more abundant in UFs than in CFs. *B*_*C*_ values and protease activity were markedly higher in upper horizons.

Thus, the activity of plant root, soil fauna and fungi increased following the long-term cessation of use of chemical fertiliser, pesticides and weed killers and deep tillage and the input mulch in UFs. These biological activities probably promote the development of soil structure from surface soil to subsoil and increase ESD. Therefore, these developments of soil structure and ESD improve the productivity of UFs on andosols in Japan.

## References

[CR1] Blake GR, Hartge KH (1986) Bulk density. In: Klute A (ed) Methods of soil analysis part 1. Physical and mineralogical methods, 2nd edn, agronomy monograph no. 9. ASA and SSSA, Madison, pp 364–367

[CR2] Blakemore LC, Searle PL, Daly BK (1981). Methods for chemical analysis of soils, New Zealand Soil Bureau Scientific Report 10A.

[CR3] Brady NC, Weil RR (2008). The nature and properties of soils.

[CR4] Briggs JM, Knapp AK (1995). Interannual variability in primary production in tallgrass prairie: climate, soil water content, topographic position, and fire as determinants of aboveground biomass. Am J Bot.

[CR5] Bullock P, Fedoroff N, Jongerius A, Stoops G, Tursina T, Babel U (1985). Handbook for soil thin section description.

[CR6] Ciarkowska K (2010). Effect of fertilization on the structure of upland grassland soil. Pol J Environ Stud.

[CR7] Climate-Data.Org (2015a) Climate-Data.org>Asia>Japan>Hokkaido>Eniwa, Climate: Eniwa. http://ja.climate-data.org/location/4194/. Accessed 24 Nov 2015

[CR01] Climate-Data.Org (2015b) Climate-Dara.org>Asia>Japan>Chiba>Narita, Climate: Narita. http://ja.climate-data.org/location/764720/. Accessed 24 Nov 2015

[CR8] Committee of Soil Environmental Analysis (1970). Methods of soil environmental analysis.

[CR9] Dathe A, Eins S, Niemeyer J, Gerold G (2001). The surface fractal dimension of the soil–pore interface as measured by image analysis. Geoderma.

[CR10] Daynes CN, Field DJ, Saleeba JA, Cole MA, McGee PA (2013). Development and stabilisation of soil structure via interactions between organic matter, arbuscular mycorrhizal fungi and plant roots. Soil Biol Biochem.

[CR11] Ersahin S, Gunal H, Kutlu T, Yetgin B, Coban S (2006). Estimating specific surface area and cation exchange capacity in soils using fractal dimension of particle-size distribution. Geoderma.

[CR13] Fitzpatrick EA (1984). Micromorphology of soil.

[CR14] Fuziwara S, Anzai T, Ogawa Y, Katoh T (2010) An encyclopedia of soil and plant nutrition version 2.0. Nobunkyo, Rural Culture Association Japan, Tokyo

[CR15] Gerasimova M, Lebedeva-Verba M, Stoops G, Marcelino V, Mees F (2010). Topsoils—mollic, takyric and yermic horizons. Interpretation of micromorphological features of soil and regoliths.

[CR16] Gerhardt RA (1997). A comparative analysis of the effects of organic and conventional farming systems on soil structure. Biol Agric Horitc.

[CR17] Hattori T, Hattori R, McLaren AD (1976). The physical environment in soil microbiology: an attempt to extend principles of microbiology to soil microorganisms. Crit Rev Microbiol.

[CR18] Hayano K (1973). A method for the determination of β-glucosidase activity in soil. Soil Sci Plant Nutr.

[CR19] Hermavan B, Cameron KC (1993). Structural changes in a silt loam under long-term conventional or minimum tillage. Soil Tillage Res.

[CR20] Hillel D (1998). Environmental soil physics.

[CR21] Hojito M, Ikeguchi A, Kohyama K, Shimada K, Ogino A, Mishima S, Kaku K (2003) Estimation of nitrogen loading in japanese prefectures and scenario testing of abatement strategies. Jpn J Soil Sci Plant Nutr 74:467–474 **(in Japanese)**. http://ci.nii.ac.jp/naid/110001754948/

[CR22] Ishii (2010) The ultimate vegetables. Natural-Seed Network, Chiba **(in Japanese)**

[CR23] Ishiyaku Publishers (2011). New food composition table in Japan.

[CR03] IUSS Working Group (2014) World reference base for soil resources 2014, International soil classification system for naming soils and creating legends for soil maps, World soil resources reports, No. 106. FAO, Rome

[CR24] Japan Meteorological Agency (2015a) Home>Data and information>Past weather data search>Average values (year and months), Average values (year and months) in Eniwa and Shimamatsu. http://www.data.jma.go.jp/obd/stats/etrn/view/nml_amd_ym.php?prec_no=14&block_no=0035&year=&month=&day=&view=p1. Accessed 24 Nov 2015

[CR02] Japan Meteorological Agency (2015b) Home>Data and information>Past weather data search>Average values (year and months), Average values (year and months) in Narita. http://www.data.jma.go.jp/obd/stats/etrn/view/nml_amd_ym.php?prec_no=45&block_no=0378&year=&month=&day=&view=p1. Accessed 24 Nov 2015

[CR25] Jenkinson DS, Ladd JN, Paul EA, Ladd JN (1981). Microbial biomass in soil: measurement and turnover, in soil biochemistry. Soil biochemistry.

[CR26] Joergensen RG, Brookes PC (1990). Ninhydrin-reactive nitrogen measurements of microbial biomass in 0.5 m K_2_SO_4_ soil extracts. Soil Biol Biochem.

[CR27] Karasawa T, Takebe M, Sato F, Komada M, Nagaoka K, Takenaka M, Urashima Y, Nishimura S, Takahashi S, Kato N (2015). Trends of lettuce and carrot yields and soil enzyme activities during transition from conventional to organic farming in an andosol. Soil Sci Plant Nutr.

[CR28] Kato H (2014) Plow pan, plowsole. In: The Editorial Committee of an Encyclopedia of Soil (ed) An encyclopedia of soil. Maruzen, Tokyo

[CR29] Kovda I, Mermut A, Stoops G, Marcelino V, Mees F (2010). Vertic features. Interpretation of micromorphological features of soil and regoliths.

[CR30] Kumazawa K (1999) Present state of nitrate pollution in groundwater. Jpn J Soil Sci Plant Nutr 70:207–213 **(in Japanese)**. http://ci.nii.ac.jp/naid/110001747123/

[CR31] Kuwada M, Shiraiwa T, Horie T (2006) Changes in total nitrogen and total carbon contents in soil and the leaf yield of a long-term unfertilized mulberry under field condition (agronomy). Jpn J Crop Sci 75:28–37 **(in Japanese)**. http://ci.nii.ac.jp/naid/110004101403/

[CR32] Ladd JN, Butler JHA (1972). Short-term assays of soil proteolytic enzyme activities using proteins and dipeptide derivatives as substrates. Soil Biol Biochem.

[CR33] Lambers H, Shane MW, Cramer MD, Pearse SJ, Veneklaas EJ (2006). Invited review, root structure and functioning for efficient acquisition of phosphorus: matching morphological and physiological traits. Ann Bot.

[CR34] Langmaack M (1999). Earthworm communities in arable land influenced by tillage, compaction, and soil. Z Ökol Natursch.

[CR35] Lee DM, Elrick DE, Reynolds WD, Clothier BE (1985). A comparison of three field methods for measuring saturated hydraulic conductivity. Can J Soil Sci.

[CR36] MAFF (Ministry of Agriculture, Forestry and Fisheries) (2015) Statistics table, statistics of vegetable production and statistics. http://www.maff.go.jp/j/tokei/kouhyou/sakumotu/sakkyou_yasai/index.html. Accessed 9 Dec 2015

[CR37] Mander U (1999). Ecological and low intensity agriculture as contributors to landscape and biological diversity. Landsc Urban Plan.

[CR38] Marschner P, Kandeler E, Marschner B (2003). Structure and function of the soil microbial community in a long-term fertilizer experiment. Soil Biol Biochem.

[CR39] Miyoshi H (1972) Consideration of the effective soil depth in good soil condition farmland for plant roots. Jpn J Soil Sci Plant Nutr 43:92–97. http://ci.nii.ac.jp/naid/110001749725/

[CR40] Morph S, Tech S (2006) Plough pan (Brit.); plough sole (Brit.); plow pan (Amer.); plow sole (Amer.); tillage pan. In: Canarache A, Vintila I, Munteanu I (eds) Elsevier’s dictionary of soil science: in English (with definitions), French, German and Spanish. Elsevier, Amsterdam, p 658

[CR41] Nagatsuka S, Tamura K (1986) An application of micropedology to the study of plant–soil system—with special reference to improvement of method for making soil thin section. Jpn J Ecol 36:163–168 **(in Japanese)**. http://ci.nii.ac.jp/naid/110001881792/

[CR42] Oades JM (1993). The role of biology in the formation, stabilization and degradation of soil structure. Geoderma.

[CR43] Oda M, Hosen Y (2011). Estimation of the origin of nitrogen in Tomato cultivated under an unfertilized condition. Jpn J Crop Sci.

[CR44] Okada M (1953). Descant of natural farming.

[CR45] Pagliai M, Vignozzi N, Pellegrini S (2004). Soil structure and the effect of management practices. Soil Tillage Res.

[CR46] Papadopoulos A, Bird NRA, Whitmore AP, Mooney SJ (2014). Does organic management lead to enhanced soil physical quality?. Geoderma.

[CR47] Plante AF, McGill WB (2002). Soil aggregate dynamics and the retention of organic matter in laboratory-incubated soil with differing simulated tillage frequencies. Soil Tillage Res.

[CR48] Pulleman M, Jongmans A, Marinissen J, Bouma J (2003). Effects of organic versus conventional arable farming on soil structure and organic matter. Soil Use Manag.

[CR49] Ranjard L, Richaume A (2001). Quantitative and qualitative microscale distribution of bacteria in soil. Res Microbiol.

[CR50] Rhoton FE, Lindbo DL (1997). A soil depth approach to soil quality assessment. J Soil Water Conserv.

[CR51] Rillig MC, Mummey DL (2006). Tansley review mycorrhizas and soil structure. New Phytol.

[CR52] Ritz K, Young IM (2004). Interactions between soil structure and fungi. Mycologist.

[CR53] Rumpel C, Kögel-knabner I (2011). Deep soil organic matter—a key but poorly understood component of terrestrial C cycle. Plant Soil.

[CR54] Saigusa M (2014) effective soil depth, effective soil layer, effective depth of soil. In: The Editorial Committee of An Encyclopedia of Soil (ed) An encyclopedia of Soil. Maruzen, Tokyo

[CR55] Sakurai M, Suzuki K, Onodera M, Shinano T, Osaki M (2007). Analysis of bacterial comminities in soil by PCR-DGGE targeting protease genes. Soil Biol Biochem.

[CR56] Sasaki H, Shibata S, Hatanaka T (1994) Method for evaluation of Japanese lawn grass (*Zoysia japonica* steud.) ecotypes for different purposes. Bull Natl Grassl Res Inst 49:17–24 **(in Japanese)**. http://ci.nii.ac.jp/naid/40002236515/

[CR57] Schollenberger CJ, Simon RH (1945). Determination of exchange capacity and exchangeable bases in soils. Soil Sci.

[CR58] Sedov S, Stoops G, Shoba S, Stoops G, Marcelino V, Mees F (2010). Regoliths and soils on volcanic ash. Interpretation of micromorphological features of soil and regoliths.

[CR59] Shoji S, Nanzyo M, Dahlgren RA (1993). Volcanic ash soils: genesis, properties and utilization.

[CR60] Silberbush M, Barber SA (1983). Sensitivity of simulated phosphorus uptake to parameters used by a mechanistic-mathematical model. Plant Soil.

[CR61] Six J, Elliott ET, Paustian K (1999). Aggregate and soil organic matter dynamics under conventional and No-tillage systems. Soil Sci Soc Am J.

[CR62] Six J, Bossuyt H, Degryze SD, Denef K (2004). A history of research on the link between (micro) aggregates, soil biota, and soil organic matter dynamics. Soil Tillage Res.

[CR63] Takayashu H (1986). Fractal.

[CR64] Tamura K, Nagatsuka S, Oba Y (1993) Effects of secondary succession on micromorphology of ando soils in Central Japan. Jpn J Soil Sci Plant Nutr 64:183–189 **(in Japanese)**. http://ci.nii.ac.jp/naid/110001751272/

[CR65] The Fourth Committee for Soil Classification and Nomenclature in the Japanese Society of Pedology (2010) Field book for describing and sampling soils, version 2.0. Hakuyusya, Tokyo

[CR66] Thomas GW, Sparks DL (1996). Soil pH and soil acidity. Methods of soil analysis, part 3, chemical methods.

[CR67] Tisdall JM, Oades JM (1982). Organic-matter and water-stable aggregates in soils. J Soil Sci.

[CR68] Truog E (1930). The determination of the readily available phosphorus of soils. J Am Soc Agron.

[CR69] Van Reeuwijk LP (2002). Procedures for soil analysis.

[CR70] Vance ED, Brookes PC, Jenkinson DS (1987). An extraction method for measuring soil microbial biomass C. Soil Biol Biochem.

[CR71] Verran J, Boyd RD (2001). The relationship between substratum surface roughness and microbiological and organic soiling: a review. Biofouling.

[CR72] Yamaki A, Kusuda T, Kagawa A, Furuno K (2013). Nitrate ion concentrations in soil water to 30 m depth in a vegetable field on the Shimousa tableland, Japan. CAFRC Res Bull.

[CR73] Yoshida M (1987) The research of potato 20th report. Physio-ecological studies of potato plant XX. On the growth and yield of no-fertilizer. Jpn J Crop Sci 27:6 **(in Japanese)**. http://ci.nii.ac.jp/naid/110008159294

[CR74] Yoshima Y (2005) What is “the unfertilized cultivation” everyone is talking about talked-about cultivation. Jpn J Modern Agric 84:292–300 **(in Japanese)**. http://ci.nii.ac.jp/naid/40006890152/

[CR75] Yoshino A (1993) Non-chemical fertilizer cultivation in vegetable upland field where long term application of compost. Jpn J Agric Tech 48:400–403 **(in Japanese)**. http://ci.nii.ac.jp/naid/40003105862/

[CR76] Young IM, Ritz K (2000). Tillage, habitat space and function of soil microbes. Soil Tillage Res.

